# Ferroptosis and Its Multifaceted Role in Cancer: Mechanisms and Therapeutic Approach

**DOI:** 10.3390/antiox11081504

**Published:** 2022-07-31

**Authors:** Heshu Chen, Chenyu Wang, Zemin Liu, Xinmiao He, Wenjie Tang, Liuqin He, Yanzhong Feng, Di Liu, Yulong Yin, Tiejun Li

**Affiliations:** 1 Hunan Provincial Key Laboratory of Animal Nutritional Physiology and Metabolic Process, CAS Key Laboratory of Agro-Ecological Processes in Subtropical Region, National Engineering Laboratory for Pollution Control and Waste Utilization in Livestock and Poultry Production, Institute of Subtropical Agriculture, Chinese Academy of Sciences, Changsha 410125, China; chenheshu@haas.cn (H.C.); wangchenyu_su@hunnu.edu.cn (C.W.); liuzemin17@mails.ucas.ac.cn (Z.L.); yinyulong@isa.ac.cn (Y.Y.); 2 Institute of Animal Husbandry, Heilongjiang Academy of Agricultural Sciences, Harbin 150086, China; 777@haas.cn (X.H.); fengyanzhong@haas.cn (Y.F.); 3 Hunan Provincial Key Laboratory of Animal Intestinal Function and Regulation, Laboratory of Animal Nutrition and Human Health, College of Life Sciences, Hunan Normal University, Changsha 410081, China; 4 Animal Breeding and Genetics Key Laboratory of Sichuan Province, Sichuan Animal Science Academy, Chengdu 610066, China; wenhan28@126.com

**Keywords:** ferroptosis, lipid peroxidation, iron, cancer, oxidative stress

## Abstract

Ferroptosis, a new type of non-apoptotic cell death modality, is different from other modes of cell death and has been primarily found in tumor cells. Previous studies have reported that ferroptosis can be triggered by specific modulators (e.g., drugs, nutrients, and iron chelators), leading to increased intracellular lipid reactive oxygen species (ROS) accumulation and iron overload. Recent reports have shown that ferroptosis at the cellular and organism levels can prevent an inflammatory storm and cancer development. Emerging evidence suggests potential mechanisms (e.g., system Xc-, glutathione peroxidase 4 (GPX4), lipid peroxidation, glutathione (GSH), and iron chelators) are involved in ferroptosis, which may mediate biological processes such as oxidative stress and iron overload to treat cancer. To date, there are at least three pathways that mediate ferroptosis in cancer cells: system Xc-/GSH/GPX4, FSP1/CoQ10/NAD(P)H, and ATG5/ATG7/NCOA4. Here, we summarize recent advances in the occurrence and development of ferroptosis in the context of cancer, the associations between ferroptosis and various modulators, and the potential mechanisms and therapeutic strategies targeting ferroptosis for the treatment of cancer.

## 1. Introduction

Ferroptosis is an iron-dependent cell death mechanism first observed in oncogenic RAS mutant cells induced by RAS-selective lethal small molecules (e.g., erastin and RLS3) [1]. It has been reported that ferroptosis differs from apoptosis, classic necrosis, autophagy, and other forms of cell death based on morphological, biochemical, and genetic criteria [1]. The main consequence of ferroptosis is cell death; it is usually accompanied by mitochondrial damage, such as the reduction or elimination of the mitochondrial cristae, rupture of the outer mitochondrial membrane, and a lack of rupture or blebbing of the plasma membrane [2]. Previous studies have reported that ferroptosis is best characterized by its ability to accumulate iron, resulting in lipid peroxidation [1]. However, this process can be blocked by ferroptosis inhibitors, such as Ferrostatin-1 (Fer-1) [1], Liproxstatin-1 (Lip-1) [3], and iron chelators [1]. Recent studies have provided new insight into the implications of ferroptosis in different pathological and physiological conditions, such as neurodegenerative diseases [4,5], ischemia/reperfusion injury [6], heart disease [7,8], and tumors [9]. To date, many related pathways have been found to mediate ferroptosis in cancer cells, including system Xc-/GSH/GPX4, FSP1/CoQ10/NAD(P)H, and ATG5/ATG7/NCOA4 pathways [10], which are considered potential therapeutic targets, especially for the treatment of cancers. However, the key mechanisms involved in the induction or suppression of ferroptosis in animals and humans, as well as the cross talk between ferroptosis and other cell death pathways (e.g., apoptosis, necrosis, and autophagy), are just beginning to be fully understood. In this review, we highlight recent advances in the remarkable regulatory versatility and potential mechanisms of ferroptosis in cancers.

## 2. The Regulatory Mechanisms of Ferroptosis

### 2.1. System Xc-/GSH/GPX4 Pathway

The system Xc-/GSH/GPX4 pathway was the first pathway identified as playing an important role in ferroptosis. Previous reports have demonstrated that ferroptosis generally leads to oxidative stress. Some small molecules and anti-carcinogens can directly or indirectly activate system Xc- to maintain the redox state of cancer cells and protect against tumor growth. Some studies have also reported that the transmembrane protein complexes, SLC7A11 and SLC3A2 and system Xc- cysteine/glutamate antiporters, can protect cells from ferroptosis-induced death [11]. The main mechanisms underlying this phenomenon include: I. Cysteine can be reduced from cystine by a neutral amino acid transporter, or via endogenous production using methionine and glucose via transsulfuration. II. GSH, as a crucial precursor of iron-sulfur and a cofactor for GPX and glutathione-S-transferases, is dependent on cystine uptake through system Xc- and plays a vital role in eliminating excess ROS. III. SLC7A11 is generally overexpressed in cell ferroptosis. IV. GPX4 is known as the main enzyme of the antioxidant system responsible for removing lipid hydroperoxides, so the lack of GPX4 activity and expression will trigger ferroptosis [12].

**Figure 1:**
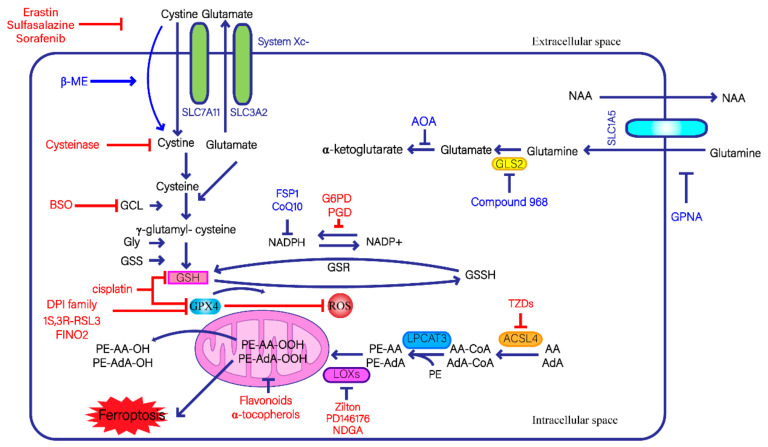
The signaling pathways and regulatory mechanisms of lipid-peroxidation-mediated ferroptosis. The lines and text in red denote ferroptosis inducers, while the lines and text in blue denote ferroptosis inhibitors. System Xc- consists of SLC7A11 and SLC3A2. SLC1A5 is a glutamine importer [62]. GSH is synthesized from Cys, Glu, and Gly in two steps: firstly, the ATP-dependent cytosolic enzyme GCL synthesizes γ-glutamyl-cysteine from Gln and Cys; secondly, GSS synthesizes GSH from γ-glutamyl-cysteine and Gly. GPX4 and GSH can prevent lipid ROS accumulation. GSH reductase utilizes NADPH to reduce GSSG to GSH. ACSL4 utilizes AA and AdA to synthesize AA-CoA and AdA-CoA. LPCAT3 catalyzes ACSL4-synthesized AA-CoA and AdA-CoA into PE species. Lipoxygenase oxidizes PE species into PUFA-OOHs. Lastly, GPX4 catalyzes the lipid hydroperoxides PUFA-OOHs into lipid alcohols PUFA-OH.

System Xc- is an antiporter that imports cystine in and exports glutamate out of cells, respectively, and has been found to play an important role in ferroptosis (Figure 1) [13]. It is known that cystine/glutamate exchange by system Xc- occurs at a 1:1 ratio [14]. High extracellular levels of glutamate may inhibit this exchange and induce ferroptosis [15]. There are high amounts of GSH and small amounts of GPX4 in the intermembrane space (IMS) of the mitochondria, which can effectively alleviate oxidative damage [16,17]. Yang et al. (2020) reported that erastin could directly bind to the mitochondrial voltage-dependent anion channels 2 and 3 (VDAC2/3) in melanoma cells and trigger ferroptosis (Table 1) [18]. Glutaminase (GLS), including its two isoforms, GLS1 in the cytoplasm and GLS2 in the mitochondria, can catalyze glutaminolysis [19]. Intriguingly, the knockdown of GLS2 (Table 2), but not GLS1, inhibited serum-dependent necrosis in mouse embryonic fibroblasts. Serum-dependent necrosis involves ferroptosis, or at least these two modes share the same mechanisms [20]. These results suggest that the mitochondria are potentially related to ferroptosis. Early studies identified VDAC2 and VDAC3 as direct erastin targets. However, it was later clarified that erastin induced ferroptosis by inhibiting SLC7A11 expression in system Xc- [21].

**Table 1:** Modulators involved in ferroptosis.

Compound/ Modulator	Target	Effect	Function	References
Erastin	VDAC2/3 and System Xc-	Induces ferroptosis	Inhibits System Xc- activity, prevents cystine import, causes GSH depletion, and destroys mitochondria	[1,18,22,23]
Sulfasalazine (SSZ)	System Xc-	Induces ferroptosis	Inhibits System Xc- activity	[1,24]
Sorafenib	System Xc-	Induces ferroptosis	Inhibits System Xc- activity	[24,25]
Glutamate	System Xc-	Induces ferroptosis	Inhibits System Xc- activity and converts into α-KG	[1,15,23,26]
IMCA	System Xc-	Induces ferroptosis	Inhibits system Xc- through the AMPK/mTOR pathway	[27]
1S,3R-RSL3, DPI19, DPI18, DPI17, DPI13, DPI12, DPI10(ML210) and DPI7(ML162)	GPX4	Induces ferroptosis	Inhibits GPX4 and causes an accumulation of lipid hydroperoxide	[9,28,29]
FINO_2_	GPX4	Induces ferroptosis	Indirectly inhibits GPX4 enzymatic function	[30]
FIN56	GPX4 and CoQ10	Induces ferroptosis	Degrades GPX4 and depletes CoQ10	[31]
BSO	GCS	Induces ferroptosis	Inhibits GCS in GSH synthesis	[32]
α-KG	Glutaminolysis	Induces ferroptosis	Inhibits the glutaminolysis pathway	[20]
Cisplatin	GPX4, GSH	Induces ferroptosis	GSH depletion and GPX4 inactivation	[33]
Lapatinib	Iron	Induces ferroptosis	Decreases ferroportin expression and increases transferrin expression	[34]
Siramesine	Iron	Induces ferroptosis	Decreases ferroportin expression and increases transferrin expression	[34]
β-ME	System Xc-	Inhibits ferroptosis	Imports cystine into cell by a transporter other than System Xc-	[1,35]
Nedd4 ubiquitylated	Mitochondria	Inhibits ferroptosis	Degrades VDAC2/3	[18]
Vitamin E (α-tocopherol, tocotrienols)	Lipid peroxidation	Inhibits ferroptosis	Reduces lipid peroxidation by inhibiting ROS production	[36]
Ferrostatin-1	Lipid peroxidation	Inhibits ferroptosis	Reduces lipid peroxidation by inhibiting ROS production	[37]
Liproxstatin-1	Lipid peroxidation	Inhibits ferroptosis	Reduces lipid peroxidation by inhibiting ROS production	[37,38]
DFO	Iron	Inhibits ferroptosis	Depletes iron and prevents the Fenton reaction	[1,25,39]
Deferiprone	Iron	Inhibits ferroptosis	Depletes iron and prevents Fenton reaction	[1,25]
PD146176	LOX	Inhibits ferroptosis	Inhibits 15-LOX	[40]
PepA-Me	Ferritin	Inhibits ferroptosis	Prevents ferritinophagy	[41]
NDGA	LOX	Inhibits ferroptosis	Inhibits pan-LOX	[40]
Thiazolidinediones	ACSL4	Inhibits ferroptosis	Inhibits ACSL4	[42]

**Table 2:** Genes and proteins identified in ferroptosis.

Gene	Protein	Function	Reference
ACSL4	Acyl-CoA synthetase long-chain family member 4	Catalyzes synthesis of long-chain polyunsaturated CoAs	[42,43]
ACSF2	Acyl-CoA synthetase family member 2	Knockdown suppresses erastin-induced ferroptosis	[1]
ATP5G3	ATP synthase F0 complex subunit C3	Knockdown suppresses erastin-induced ferroptosis	[1]
AIFM2	apoptosis-inducing factor mitochondria-associated 2	Inhibits ferroptosis elicited by GPX4 deletion	[44,45]
ATG5	Autophagy-related gene 5	Inhibits ferritinophagy	[10]
ATG7	Autophagy-related gene 7	Inhibits ferritinophagy	[10]
CS	Citrate synthase	Knockdown inhibits erastin-induced ferroptosis	[1]
FBXL5	Leucine-rich repeat protein 5	A key regulator of iron homeostasis	[46,47]
GCLC	Glutamate-cysteine ligase	GSH synthesis	[19,48]
GLS2	Glutaminase 2	GSH synthesis	[20]
GSS	Glutathione synthetase	GSH synthesis	[19]
GPX4	Glutathione peroxidase 4	Lipid ROS scavenger	[9,49]
IREB2	Iron-responsive element binding protein 2 Lipoxygenases	Key regulator of iron homeostasis Involved in PUFAs peroxidation	[1]
LPCAT3	Lysophosphatidylcholine acyl-transferase 3	Involved in PE biosynthesis	[36]
NCOA4	Nuclear receptor coactivator 4	Mediates ferritinophagy contributing to ferroptosis	[50,51]
PEBP1	Phosphatidylethanolamine binding protein 1	PUFA-PE and 15-HpETE-PE accumulation	[52]
RPL8	Ribosomal protein L8	Knockdown suppresses erastin-induced ferroptosis	[1]
SLC7A11	Subunit solute carrier family 7 member 11	Cystine/glutamate antiporter	[23]
SAT1	Spermidine/spermine N1-acetyltransferase 1	Involved in lipid peroxidation	[35]
TTC35	Tetratricopeptide repeat domain 35	Knockdown suppresses erastin-induced ferroptosis	[1]
TfR1	Transferrin receptor 1	Imports extracellular Fe^3+^ into the cell	[53]
DMT1	Divalent metal transporter 1	Mediates the release of Fe^2+^ from the endosome into the labile iron pool	[54]
FPN1	Ferroportin 1	The only mammalian non-heme iron exporter	[55,56]
Hepc	Hepcidin	Regulates ferroportin-mediated iron export	[57]
IRP	Iron regulatory protein	Regulates cellular iron metabolism by binding to IREs, including IRP1 and IRP2	[58]
TP53	Tumor protein 53	Inhibits SAT1, DPP4 activity, CDKN1A/p21, and System Xc-	[59,60,61]

GSH has been found to significantly attenuate oxidative stress and apoptosis [63]. Several studies have shown that GSH can also eliminate lipid peroxidation and suppress ferroptosis [49]. In mammals, the GPX family of proteins, including GPX1–8, has been found to play vital roles in the response to oxidative stress. Among these, only GPX4, a GSH-dependent enzyme that effectively decreases lipid hydroperoxide contents, plays a crucial role in ferroptosis [64]. Some studies have reported that RAS-selective lethal small molecule 3 (RSL3) directly inhibits GPX4 by targeting the alkylation of the catalytic selenol [9]. However, only 1S, 3R-RSL3, one of the four RSL3 diastereomers, can induce ferroptosis by binding to GPX4 [9]. Aside from GPX4, it has been found that 1S, 3R-RSL3 can also bind to three other selenoproteins including SELS, SELM, and EPT1 [65]. Recently, Vuckovic et al. (2020) discovered that protein 14-3-3e is a key factor in the binding between 1S, 3R-RSL3 and GPX4 [66]. After binding to GPX4, RSL3 directly inactivates GPX4 to induce lipid ROS accumulation and ferroptosis [9]. GSH and GPX4 can convert toxic lipid hydroperoxides into non-toxic lipid alcohols and suppress lipid peroxidation, thereby limiting ferroptosis [49]. Once GSH is depleted, the iron-dependent accumulation of lipid hydroperoxides results in an iron-catalyzed Fenton reaction, which can produce ROS, resulting in the accumulation of lipid hydroperoxides and ferroptosis [67]. The equilibrium between glutathione-S-S-glutathione (GSSG) and GSH can be exploited to maintain redox homeostasis [68]. It is well known that GSSG is a GSH disulfide that can be reduced to GSH by glutathione-disulfide reductase (GSR) using NADPH. GSH assists the production of GPX enzymes to reduce hydrogen and lipid peroxides, thereby inhibiting ferroptosis [69]. NADPH can predict sensitivity to ferroptosis, whereas NADPH levels are inversely related to the sensitivity to ferroptosis inducers [70]. Some studies have confirmed that the knockdown of glucose-6-phosphate dehydrogenase (G6PD) and phosphoglycerate dehydrogenase (PGD) limited the production of NADPH and prevented erastin-induced ferroptosis [1].

### 2.2. FSP1/CoQ10/NAD(P)H Pathway

Recently, flavoprotein apoptosis-inducing factor mitochondria-associated 2 (AIFM2) has been found to play a key role in preventing ferroptosis [44]. AIFM2, now renamed ferroptosis suppressor protein 1 (FSP1), can inhibit lipid peroxide accumulation and protect against ferroptosis caused by GPX4 deletion (Figure 1) [44]. Doll et al. (2019) found that cancer cell lines with FSP1 knocked out were more sensitive to ferroptosis inducers which were rescued by FSP1 overexpression. Further studies have shown that FSP1 can prevent lipid peroxidation via the NADH-dependent coenzyme Q (CoQ) oxidoreductase pathway once ferroptosis occurs [71]. Bersuker et al. (2019) reported that FSP1 acts as an NADH-dependent CoQ oxidoreductase and indirectly affects ferroptosis [45]. FSP1 blocks and attenuates the production of ferroptotic signals by controlling the reduction in CoQ10 in the non-mitochondrial compartment [71]. Moreover, blocking FSP1 or depleting CoQ10 can significantly increase sensitivity to ferroptosis. FSP1 functions independently of cellular GSH levels or GPX4 activity [44]. Therefore, FSP1/CoQ10/NAD(P)H, a new surveillance pathway distinct from the GSH/GPX4 protective pathway, was identified. To date, there are few reports on the inhibitors and inducers of the FSP1/CoQ10/NAD(P)H pathway. Moreover, the expression of FSP1 was found to be suppressed in many human cancers; therefore, further studies should be conducted to confirm the effects of this phenomenon.

### 2.3. Lipid-Peroxidation-Mediated Mechanism

Lipid metabolism also plays an important role in ferroptosis. Acyl-CoA synthetase long-chain family member 4 (ACSL4) plays an important role in lipid droplet biogenesis (Figure 1) [72]. ACSLs are a family of enzymes comprising five isoforms: ACSL1, ACSL3, ACSL4, ACSL5, and ACSL6 [73]. Among them, ACSL4, the preferred long-chain polyunsaturated fatty acid, has been found to sensitize breast cancer cells towards ferroptosis [42]. ACSL4 utilizes arachidonic acid (AA) and adrenic acid (AdA) to synthesize AA-CoA and AdA-CoA [42,43], respectively. Lysophosphatidylcholine acyl-transferase 3 (LPCAT3) has been identified as an enzyme that catalyzes the conversion of long-chain PUFA-CoAs into long-chain phosphatidylethanolamine (PE)-based substrates, which can catalyze ACSL4-synthesized AA-CoA and AdA-CoA into PE species (PE-AA and PE-AdA, respectively) [36]. After compounding PE species, lipoxygenase oxidizes PE-AA and PE-AdA into PE-AA-OOH and PE-AdA-OOH. Lastly, GPX4 catalyzes the conversion of lipid hydroperoxides PUFA-OOHs (such as PE-AA-OOH and PE-AdA-OOH) into lipid alcohols PUFA-OH (such as PE-AA-OH and PE-AdA-OH), thereby inhibiting ferroptosis.

PUFA oxidation by lipoxygenases is necessary for ferroptosis [74]. Lipoxygenases (LOXs) are a family of enzymes comprising six isoforms in humans: 3-LOX, 5-LOX, 12-LOX-1, 12-LOX-2, 15-LOX-1, and 15-LOX15-2 [75]. Among these, 15-LOX has been previously found to be involved in ferroptosis [74]. The conversion of eicosatetraenoic-acid-phosphatidylethanolamine (ETE-PE) to 15-hydroperoxyeicosatetraenoic-phosphatidylethanolamines (15-HpETE-PE) can be catalyzed by 15-LOX with non-heme iron [76]. Insufficient levels of GPX4 or GSH result in the inability in the cell to reduce newly formed 15-HpETE-PE, causing its accumulation and eventually leading to ferroptosis [76]. This indicates that 15-HpETE can be considered a ferroptosis signal. Moreover, RSL-3 may affect lipid peroxidation in the liver via a 12/15-Lox-AIF-related pathway. The activation of 12/15-LOX aggravates endoplasmic reticulum stress, inflammation, liver steatosis, and liver damage; it contributes to metabolic-associated fatty liver disease (MAFLD) by iron chelators. However, some reports have also found that LOX inhibitors with radical-trapping antioxidants (RTAs) activity can suppress ferroptosis, whereas LOX inhibitors without RTAs activity (e.g., CAY10649 and CJ-13610) cannot suppress ferroptosis [40]. Thus, LOX may not necessarily be required to catalyze the oxidation of AA during ferroptosis; this requires further study.

### 2.4. Iron-Mediated Mechanism

**Figure 2:**
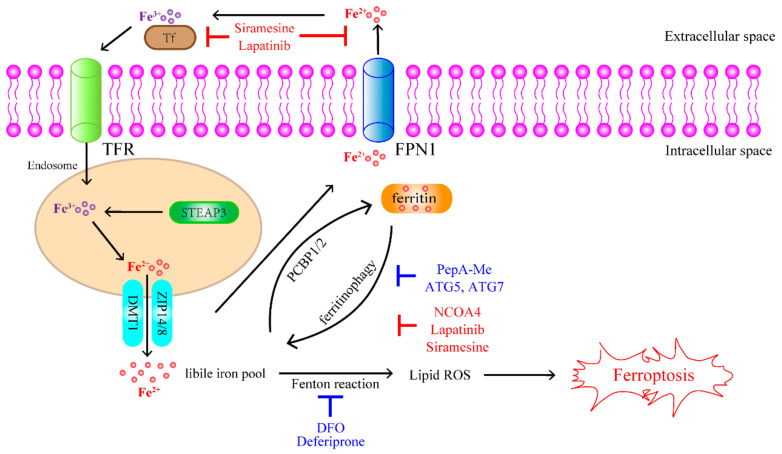
The mechanisms of iron-mediated ferroptosis. Fe^3+^ is imported into the cell via the TfR1 membrane protein and reduced to Fe^2+^ in endosome. DMT1 or ZIP14/8 transports Fe^2+^ from the endosome into the LIP. The Fe^2+^ in the LIP then undergoes any of the following three steps: (i) Fe^2+^ is oxidized by Fpn1 and is exported out of the cell; (ii) the excess Fe^2+^ is stored in ferritin; (iii) Fe^2+^ is utilized in the Fenton reaction, causing the accumulation of ROS, thereby inducing ferroptosis.

Excessive iron levels contribute to ferroptosis by catalyzing the production of lipid hydroperoxides via the Fenton reaction. Consequently, both iron import and export affect the sensitivity of cells to ferroptosis. Membrane protein transferrin receptor 1 (TfR1) imports extracellular transferrin-bound iron (TBI) (Fe^3+^) into the cell through endocytosis of the TBI-TfR1 complex, which is involved in ferroptosis [53]. TfR1 has been identified as the antigen of the 3F3 ferroptotic membrane antibody (3F3-FMA), a selective ferroptosis antibody. Therefore, TfR1 is a ferroptosis marker that provides new insights into the mechanism of iron mobilization during ferroptosis [77]. Moreover, TfR1 is abundantly expressed and is involved in the progression of several cancers, suggesting that it may be a potential therapeutic target for cancer through the iron metabolism pathway in ferroptosis [78]. Six transmembrane epithelial antigens of the prostate 3 (STEAP3) protein, a member of the STEAP family, can reduce Fe^3+^ to Fe^2+^ [79]. Endosome inhibitors, such as pepstatin A-methyl ester (PepA-Me), act as ferroptosis reducers in the cytoplasm [41]. Divalent metal transporter 1 (DMT1) or ZRT/IRT-like protein (ZIP) 14/8 mediates the release of Fe^2+^ from the endosome into the labile iron pool in the cytoplasm [53,54]. Poly rC-binding protein 2 (PCBP2) can directly interact with DMT1 or Fpn1 to bind iron and promote its transfer to the cytosol or extracellular space [53,80]. Iron chelators may prevent Fe^2+^ accumulation by interrupting the Fenton reaction, thereby suppressing ferroptosis (Figure 2).

Ferritin is a protein that stores excess iron [81]. PCBP1 binds to cytosolic iron and delivers it to ferritin for storage [53]. Moreover, the autophagic degradation of ferritin has been implicated in ferroptosis. Several reports have demonstrated that preventing the autophagic degradation of ferritin is important for intracellular iron homeostasis, reducing the accumulation of lipid ROS, which can trigger ferroptosis [50]. For example, it has been found that autophagy-related gene 5 (ATG5) and autophagy-related gene 7 (ATG7), which are involved in the inhibition of ferritinophagy, can limit ferroptosis [82]. Knockdown of nuclear receptor coactivator 4 (NCOA4), a cargo receptor for ferritinophagy, was found to suppress intracellular Fe^2+^ levels, lipid peroxidation, and ferroptosis (Table 2). These results suggest that the ATG5-ATG7-NCOA4 pathway can induce ferroptosis by increasing unstable cellular iron levels [10]. In addition, the expression of iron-responsive element binding protein 2 (IREB2), a key regulator of iron metabolism, decreases the sensitivity of cells to ferroptosis (Table 2) [1].

In general, two pools of iron are believed to participate directly in ferroptosis. One is the catalytic center of non-heme iron proteins (e.g., LOXs, a family of iron-containing enzymes, particularly 15-LOX), which causes lipid peroxidation through enzymatic action. The other is Fe^2+^ from the cytosolic labile iron pool that participates in the Fenton reaction, a non-enzymatic reaction. It has been reported that 15-LOX contains mononuclear iron centers and can induce ferroptosis; thus, the iron centers of LOXs may catalyze the formation of 15-HpETE-PE, whereas Fe^2+^ from the labile iron pool participates in its decomposition. There are many overlaps between non-enzymatic and enzymatic lipid oxidation products, especially the latter, which are found in other cell death processes. Finding this relationship is not easy, but it is worth exploring in developing novel therapeutics.

### 2.5. Cross Talk between Mitochondrial Function and Ferroptosis

Recent studies have reported that the mitochondria play a crucial role in ferroptosis. For instance, the number of mitochondria sharply increased, and the cristae and the morphologies of the mitochondrial cristae and membrane are obviously changed in ferroptosis cells [1,83]. The ultrastructural feature of ferroptosis is distinctly altered mitochondrial morphology, which distinguishes it from the classic morphological alterations associated with apoptosis, necrosis, or autophagy [1]. Impairment of mitochondrial morphology and function is a major hallmark of ferroptosis and mitochondrial electron transport is one of the major sources of ROS production. Exponentially increasing ROS levels induce cell death and mitochondrial hyperpolarization during the initial course of cell death, but induce mitochondrial depolarization at the final stages of cell death [83]. Iron metabolism can be regulated by the mitochondria, which are unique organelles involved in heme synthesis [50]. Characteristic cytological changes during ferroptosis include decreasing or vanishing mitochondria cristae, the rupture of the outer mitochondrial membranes, and the condensation of the inner membranes [12].

Hydrogen peroxide and superoxide are produced by the mitochondria, and are exacerbated with increasing mitochondria [84]. Hinder et al. (2021) investigated superoxide production in mitochondria and found that antioxidative diphenylamine compounds could prevent ferroptotic cell death by protecting mitochondrial function and inhibiting ROS production [85]. Hydrogen peroxide can use aquaporins (AQPs) such as AQP3, AQP4, and AQP8 to pass through cell membranes more quickly than diffusion. The accumulation of hydrogen peroxide could accept the electrons of transition metals, such as Cu^2+^ and Fe^2+^, to generate more reactive hydroxyl radicals. Free radicals can induce lipid peroxidation and damage the mitochondrial membranes [86]. Dihydroorotate dehydrogenase, an enzyme on the mitochondrial inner membrane, can reduce ubiquinone to ubiquinol, thereby inhibiting ferroptosis in the mitochondria, unlike GPX4 and FSP1. Increased glutaminolysis leads to sensitizing ferroptosis via cysteine deprivation or by inducers inhibiting xCT, whereas the blockade of glutaminolysis inhibits ferroptosis [87]. Mitochondrial pyruvate carrier1 (MPC1) is a tumor suppressor that represses the Warburg effect, cancer cell growth, stemness, and epithelial-mesenchymal transition (EMT) [88]. A study showed that the suppression of MPC1 expression changed metabolic and EMT traits and increased mitochondrial ROS and lipid peroxidation, inducing ferroptosis in cancer cells [89]. Tumor cells are sensitive to MPC1 expression, because of their high intracellular iron levels and increased oxidative stress compared to normal cells. However, different tumors show different sensitivities towards ferroptosis, depending on their differentiation stage, gene expression, and metabolism [90]. Mitochondria provide specific lipid precursors necessary for ferroptosis via fatty acid metabolism and glutaminolysis [2,87]. Furthermore, lipid peroxides produced in vitro by mitochondria can cause mitochondrial lipid peroxidation and damage through oxidative stress. This disrupts the regulation of iron homeostasis by mitochondria, eventually leading to the development of ferroptosis [91]. Fe^3+^-transferrin forms endosomes via clathrin-dependent endocytosis. Fe^3+^ is subsequently reduced to Fe^2+^ through the metal reductase step and transported into the cytoplasm by a divalent metal transporter [92]. Gaschler et al. (2018) investigated the molecular mechanism underlying the action of ferrostatin-1 and found that ferrostatins accumulated in the lysosomes, mitochondria, and the endoplasmic reticulum, but the lysosomes and mitochondria were not required for the effective suppression of ferroptosis [93].

### 2.6. Other Regulatory Mechanisms

Fer-1 serves as a lipid ROS scavenger under oxidative stress conditions and inhibits ferroptosis by impairing lipid metabolism [1]. Fer-1 contains only one additional NH2 group compared to ethyl 4-cyclohexylamino benzoate. However, when co-incubated with cells undergoing erastin-induced ferroptosis, Fer-1, not 4-cyclohexylamino benzoate, completely abolished ferroptosis [38]. This indicates that O-phenylenediamine is a prerequisite pharmacophore for Fer-1 activity. Similar to Fer-1, Lip-1 is a ferroptosis inhibitor with RTA activity. The intermediate aminyl radical of Lip-1 reacts with the second peroxyl radical to form nitroxide, which can catalytically capture trapping radicals, thereby inhibiting ferroptosis [37].

The tumor suppressor protein p53 (TP53) has multiple anti-cancer functions and plays a vital role in responses to several mechanisms involved in cell death [94]. Previous studies have reported that p53 can limit ferroptosis via lipid metabolism pathways that promote the expression of cyclin-dependent kinase inhibitor 1A (CDKN1A) (which encodes p21), as well as by directly blocking dipeptidyl peptidase 4 (DPP4) activity [59,60]. Recently, Chu et al. (2019) reported a 12-LOX-1-mediated, p53-dependent ferroptosis pathway. In this pathway, p53 first inhibits SLC7A11 transcription and then directly activates 12-LOX-1 function without ACSL4, resulting in 12-LOX-1-dependent ferroptosis upon ROS-induced stress [61]. Different functions of p53 have been reported in different cancer cell lines during ferroptosis; thus, it is necessary to further elucidate the accurate mechanisms and application of p53-related ferroptotic processes in cancer therapy.

## 3. Modulators That Can Induce or Inhibit Ferroptosis

Since ferroptosis is regarded as a type of iron-dependent non-apoptotic cell death, it plays a crucial role in preventing cancers and other diseases. Recently, many studies have reported that various modulators, including drugs, nutrients, and iron chelators, can trigger or inhibit ferroptosis via several common regulatory sites, such as the Xc- and GSH/GPX4 systems, lipid peroxidation, and iron [95]. We summarized these modulators in Table 1 in detail.

### 3.1. Drugs 

Erastin was first identified in 2003, as an inducer of ferroptosis in human foreskin fibroblasts (BJeLR) with engineered mutant Ras oncogene expression [22], which was used to decrease glutathione levels by directly inhibiting system Xc- activity (Table 1) and inducing ferroptosis [1]. Several modulators have been used to inhibit erastin-induced ferroptosis. For example, β-mercaptoethanol (β-ME) can generate mixed disulfides that can be absorbed by other transporters to suppress ferroptosis. This pathway avoids the requirement for system Xc−, thereby inhibiting ferroptosis [35]. Neuronal precursor-cell-expressed developmentally downregulated 4 (Nedd4) ubiquitination directly recognizes and degrades VDAC2/3 to negatively regulate erastin-induced ferroptosis [18]. Furthermore, the knockdown of acyl-CoA synthetase family member 2 (ACSF2), ATP synthase F0 complex subunit C3 (ATP5G3), citrate synthase (CS), ribosomal protein L8 (RPL8), and tetratricopeptide repeat domain 35 (TTC35) has been reported to suppress erastin-induced ferroptosis [1] (Table 2).

The function and efficacy of sulfasalazine (SSZ), sorafenib, and cisplatin are similar to those of erastin (Table 1). SSZ induces ferroptosis by blocking system Xc- activity [96], whereas the tyrosine kinase inhibitor, sorafenib, induces ferroptosis with a positive correlation between its ferroptotic potency and that of erastin [24]. However, cisplatin has been reported to induce ferroptosis by inhibiting GSH and GPX4 in A549 and HCT116 cells [33] (Table 1).

Buthionine sulfoximine (BSO) is a GSH biosynthesis inhibitor, that catalyzes the rate-limiting first step of GSH biosynthesis by directly inhibiting γ-glutamylcysteine synthetase (GCS) [32], thereby increasing intracellular lipid ROS levels and triggering ferroptosis [97]. The oxidation of phosphatidylethanolamine-species (PE-species) by LOXs can be prevented by PD146176 (a 15-LOX inhibitor) and nordihydroguaiaretic acid (NDGA, a pan-LOX inhibitor) [40] (Table 1). Phosphatidylethanolamine binding protein 1 (PEBP1), a scaffold protein inhibitor of protein kinase cascades, acts as a 15-LOX-1/2 specific rheostat, making free AA catalytic to polyunsaturated fatty acid phosphatidylethanolamines (PUFA-PE), leading to the formation of 15-HpETE-PE, which can induce ferroptosis [52].

Thiazolidinediones (TZDs), including rosiglitazone, pioglitazone, and ciglitazone, are a class of peroxisome proliferator-activated receptor gamma (PPAR-γ) agonists that selectively inhibit ACSL4 [42] (Table 1). ACSL4 can increase the incorporation of PUFAs and the lipid peroxidation required for ferroptosis [98].

### 3.2. Nutrients

Vitamin E, a chain-breaking antioxidant, can act as an RTA to protect cells from lipid peroxidation and compensate for the loss of GPX4 in certain tissues [9,99]. Compounds of the vitamin E family were found to suppress ferroptosis by competing for the PUFA substrate-binding sites of LOX [36]. α-tocopherols, some of the most biologically active members of the vitamin E family, are lipophilic antioxidants that effectively inhibit ferroptosis [36]. Baicalein, a flavonoid obtained from the roots of *Scutellaria* species, has long been known to possess antioxidant activity and attenuate oxidative cell death [100]. A recent study showed that baicalein inhibits erastin-induced ferroptosis by limiting lipid peroxidation in pancreatic cancer cells [101]. Ferroptosis has been shown to be affected by cysteine and glutamate levels [23]. Under cysteine starvation conditions, intracellular glutamate is converted into the final product of the glutamine decomposition pathway, α-ketoglutarate (α-KG). α-KG contributes to the formation of lipid ROS, thereby inducing ferroptosis [20]. Aminooxyacetic acid (AOA) is a pan-transaminase inhibitor that suppresses ferroptosis by inhibiting the glutaminolysis pathway and the formation of α-KG, indicating that α-KG is required for ferroptosis [87] (Table 1). Glutamine is imported into cells via a glutamine importer, accompanied by the efflux of neutral amino acids (NAAs) [62]. L-γ-glutamyl-p-nitroanilide (GPNA) suppresses glutamine importation by inhibiting glutamine importer activity, thus repressing ferroptosis [87]. Compound 968, a GLS inhibitor, inhibits glutamate and prevents ferroptosis [102]. Luo et al. (2018) found that miR-137 regulates ferroptosis by directly targeting SLC1A5 in melanoma cells [26].

### 3.3. Iron Chelators

Although ferroptosis can be prevented by iron chelators, it is not possible to inhibit ferroptosis using well-known small-molecule inhibitors of apoptosis, necrosis, or autophagy [1,103]. Iron chelators, including deferoxamine (DFO) and deferiprone (DFP), act as iron-depleting and ferroptosis-suppressing modulators [25,104] (Table 1). DFO is endocytosed and has been found to accumulate in lysosomes, subsequently interrupting the Fenton reaction by sequestering ferric ions and preventing ROS production [39]. It has been found that lipid peroxide formation was significantly decreased upon the addition of DFO, abolishing erastin-induced ferroptosis [1].

Treating of cells with erastin and RSL3-induced ferroptosis with PepA-Me has been shown to increase ferritin protein expression levels and reduce iron concentration, indicating that PepA-Me may prevent ferroptosis by blocking the autophagic degradation of ferritin [41]. Moreover, lapatinib, a kinase inhibitor, and siramesine, a lysosomotropic modulator, have been reported to induce ferroptosis by increasing iron levels, reducing ferroportin expression, and increasing transferrin expression [34] (Table 1).

### 3.4. Oncogenic Small Molecules

RSL3 was first discovered in a high-throughput small-molecule-screening study, where it was found to eliminate BJeLR cells in a non-apoptotic manner [29]. In addition to RSL3, DPI7 and DPI10 (also known as ML162 and ML210, respectively) have been identified as small molecules that can trigger ferroptosis via synthetic lethal screening [1,28]. In a subsequent study, other DPI family members, namely DPI19, DPI18, DPI17, DPI13, and DPI12, were found to directly inhibit GPX4 activity and lead to the accumulation of lipid peroxides, inducing ferroptosis [9] (Table 1).

### 3.5. Other Modulators

Fer-1 and Lip-1 are excellent radical-trapping antioxidants that can inhibit ferroptosis [37]. Since 2016, FIN56 (Table 1), which was derived from CIL56 and first reported as a new inducer of ferroptosis, has been proposed as an agent to suppress ferroptosis [31]. FIN56 induces ferroptosis through two distinct pathways. First, FIN56 promotes the degradation of GPX4 post-translational protein. Second, FIN56 activates squalene synthase to suppress the non-steroidogenic metabolite CoQ10 in the mevalonate pathway [70]. However, until now, it has been unclear what protein is degraded by GPX4 and the mechanism of its degradation. Unlike FIN56, the endoperoxide FINO_2_ indirectly inhibits the enzymatic function of GPX4 and induces ferroptosis through a combination of direct iron oxidation [30]. This result opens up a new avenue for the study of ferroptosis, that is, endoperoxides can initiate a multipronged ferroptotic mechanism. Recently, Zhang et al. (2020) found that the benzopyran derivative 2-imino-6-methoxy-2H-chromene-3-carbothioamide (IMCA) can induce ferroptosis mediated by system Xc- through the AMPK/mTOR pathway in colorectal cancer cells [27].

## 4. Ferroptosis in Cancer Therapeutics

### 4.1. Ferroptosis in Cancer

Traditional cancer therapies include radiotherapy and chemotherapy. However, radiation/chemotherapy resistance and adverse effects are important hurdles that must be addressed [105]. Previous studies have demonstrated a link between ferroptosis and cancer [103]. Cancer cells are susceptible to ferroptosis. This is mainly because cancer cells are metabolically active, and most are in a state of oxidative stress, easily producing a large number of ROS in cells and inducing ferroptosis. Numerous cancer-relevant genes and signaling pathways have been shown to regulate ferroptosis [106]. It has been found that cancer cells often need to consume large amounts of iron [107], which may make them more sensitive to ferroptosis. In the case of breast cancer, xCT determines the expression of GPX4, which is strongly upregulated compared to that in normal cells, so that breast cancer cells could become resistant to oxidative-stress-inducing drugs. Erastin depletes GPX4 and GPX1 levels in breast cancer cells by inhibiting xCT-dependent extracellular reduction [108]. Therefore, erastin or RSL3-induced lipid peroxidation and ferroptosis sensitivity can inhibit the proliferation of breast cancer cells.

Recent studies have focused on the role of ferroptosis in cancers. The FDA has approved several drugs, including sorafenib, SAS, and altertamin [109]. However, several unresolved issues regarding ferroptosis still exist, such as robust ferroptosis markers in vivo, specific genetic background in different cancers, and the potential adverse effects of ferroptosis-related anti-tumor drugs. To assess and predict the effects of drugs related to ferroptosis-targeted therapy, researchers need to identify biomarkers in different tumors, such as multiple ferroptosis-associated oncoproteins, tumor suppressors, and oncogenic signal transduction pathways [11]. For example, a TGGA analysis of laryngeal cancer revealed that CDKN2A has the highest mutation frequency among the top significant differential genes that IFNG significantly correlated with tumor stage and the degree of tumor differentiation [110]. In ovarian cancer, the expression levels of ferroptosis genes are significantly correlated with patient prognosis. After single-factor Cox and LASSO analyses, eight lncRNAs from the screened ferroptosis-related genes were found [111]. Several studies have suggested that lncRNAs are important in ferroptosis regulation. An example is metallothionein 1D pseudogene, which regulates erastin-induced ferroptosis via Nrf2 [112]. These findings suggest that ferroptosis biomarkers differ from those in tumors, and further studies are needed to explore the mechanisms involved.

Ferroptosis plays a dual role in tumor promotion and suppression. In summary, multiple cancer-related signaling pathways can regulate the onset of ferroptosis in cancer cells, such as p53 and BAP1. At the same time, cancer cells produce ROS in large quantities and have a high metabolism, making them more susceptible to ferroptosis. Thus, ferroptosis is always studied as a key tumor suppression mechanism. However, a few studies have also indicated that ferroptosis promotes tumorigenesis. Dai et al. (2020) found that high-iron diets or depletion of Gpx4 promoted hydroxyguanosine 8 release and activated the TMEM173 pathway and activated the DNA damage pathway in pancreatic ductal adenocarcinoma. Because of the DNA damage, macrophages were recruited to the pancreas, thus promoting the development of tumors [113]. Macrophages infiltrating into tumor tissue are susceptible to tumor-induced cytokines and become tumor-associated macrophages (TAM). These cells have an important role in disrupting adaptive immune responses and promoting tumor growth and progression [114]. In endometriosis, some stromal cells of the cyst walls underwent ferroptosis; however, endometrial stromal cell ferroptosis induced the production of angiogenic inflammatory and growth cytokines [115]. From these studies, it can be found that ferroptosis inhibits cancer development on the one hand and on the other hand leads to inflammation and cytokine secretion, thus promoting tumor progression. Thus, research into ferroptosis is still in the stage of exploration and refinement and most research is focused on cancer cell lines; there are only a few clinical studies to date.

### 4.2. Ferroptosis in Cancer Cell Lines Therapies

In tumor cells models, therapy-resistant or persistent cancer cells are more susceptible to ferroptosis inducers; therefore, many types of modulators have been used to study ferroptosis for the prevention and treatment of cancer, indicating the possibility of a close association between ferroptosis and cancer.

The development of ferroptosis-induction-based cancer therapies has been extensively discussed. Several strategies, such as delivering iron, peroxides, and other toxic cargoes, still have many problems; therefore, researchers need to develop ferroptosis-targeted approaches. As shown in Table 1, there are currently five key targets that play crucial roles in ferroptosis: system Xc-, GPX4, lipid peroxidation, LOX, and iron. System Xc- has been consistently reported as a potential target in cancer treatment [116]. Diffuse large B-cell lymphoma cells have been discovered to be more susceptible to erastin-induced ferroptosis than other types of tumor cells [9]. The low-dose ferroptosis inducer erastin could significantly enhance the anti-cancer activity of chemotherapeutic drugs (e.g., cytarabine/ara-C and doxorubicin/adriamycin), showing synergistic anti-tumor effects [117]. The combination of erastin and cisplatin therapy has also been found to have notably enhanced anti-cancer activity [33]. In addition to erastin and the widely used anti-cancer drugs SSZ and sorafenib, other inhibitors of system Xc-, such as β-ME, have potential uses in the development of novel anti-cancer drugs.

Drug-resistant cancer cells have shown characteristics of the apoptotic cascade, one of the main limitations of current cancer treatment. Another major limitation in various human cancers is their resistance to anti-cancer drugs, which can weaken the efficiency of chemotherapy by changing cancer characteristics to evade stable or complete drug response [118,119]. Poor clinical outcomes in patients with cancer are always caused by mesenchymal traits acquired by cancer cells. These traits enhance cell invasion and migration, making it difficult for traditional drugs and therapies to clear cancer cells [120]. A recent report demonstrated that cancer cells including melanoma, epithelial-derived carcinomas, and prostate cancer, display vulnerability to ferroptosis induced by the lipid peroxidase pathway [121]. Erastin, a ferroptosis-inducing drug, can directly target the resistance escape route of melanoma dedifferentiation, thereby improving the therapeutic effect on melanoma [122]. It has also been reported that the GPX4 inhibitors RSL3 and ML210 can disrupt the antioxidant system [89] and ferroptosis in many drug-tolerant cancer cells (e.g., melanoma, breast, lung, and ovarian cancer cells) in vitro and prevent tumor relapse [123]. Increased cell death and decreased cell viability were found in erlotinib-tolerant persistent head and neck cancer cells compared to their parental cells after treatment with ferroptosis inducers of RSL3 and ML210 [89]. These results indicate that ferroptosis might be a better target to overcome therapy resistance in cancer cells aside from traditional methods based on driver oncogenes and resistance mutations.

It has been reported that system Xc- deletion SLC7A11, which induces ferroptosis, can inhibit pancreatic tumor growth [124]. Metformin, a potential anti-cancer agent, induces ferroptosis in breast cancer by reducing the protein stability of SLC7A11 [125]. In a recent study, it was found that clear cell carcinoma is sensitive to GPX4-inhibition-induced ferroptosis [126]. In another study, sensitivity profiling revealed that kidney and leukemia cells were sensitive to GPX4-regulated ferroptosis [9]. Hepatocellular carcinoma is usually treated with sorafenib via ferroptosis induction [127]; however, some hepatoma cell lines are not sensitive to sorafenib-induced ferroptosis. Wang et al. found that the combination of sorafenib and RSL3 significantly promoted ferroptosis and increased ectopic levels of iron and lipid peroxides by inhibiting the Nrf2/GPX4 axis in sorafenib-resistant hepatoma cells [128]. In addition to directly affecting GPX4 by suppressing lipid peroxidation, metabolic pathways were also found to elicit the same effect by regulating glutathione levels. GSH levels were improved in different cancer cells and could provide a protective effect on cells by binding to different carcinogenic electrophiles [129]. To date, only a few drugs (e.g., BSO) directly or indirectly regulate GSH levels to treat cancer. However, some studies suggested that it would be worthwhile to use modulators to regulate GSH levels in combination with other anti-cancer drugs. The inhibition of LOX abrogates metastasis and enhances drug efficacy, wherein LOX signatures have been associated with poor prognosis in patients with pancreatic cancer [130]. These results suggest that LOX expression may serve as a potential prognostic marker for pancreatic cancer. As such, the LOX family of proteins may have potential use as a marker or adjuvant drug in the treatment of other types of cancers.

In vitro and in vivo studies have demonstrated that some aggressive tumors, such as neuroblastomas, are sensitive to iron chelation therapy using DFO [131]. These studies have shown that numerous cancer cells are more sensitive to iron chelators than normal cells. The expression of TFR1, ferritin, and ferroportin, which participate in ferroptosis, can also directly drive tumor growth [107]. Chen et al. (2016) found that ACSL4 is overexpressed in several types of cancers, including brain, colorectal, lung, breast, prostate, and liver cancer [132]. ATG proteins have also been suggested as potential prognostic markers or targets in colorectal and gastric cancer [133,134]. Mutations in the TP53 protein have become the cornerstone of various translational efforts to validate its clinical use for the diagnosis, prognosis, and treatment of cancers. GLS2, FBXL5, NCOA4, and SAT1 proteins have also been repeatedly reported as therapeutic targets in cancer and have been used in multi-angle evaluation systems for preclinical investigations [48,51,135,136,137].

Many modulators that can induce or inhibit ferroptosis have not yet been reported or are rarely reported in terms of their use in cancer drug discovery and treatment, suggesting that there are many potential opportunities for discovering new and specific treatments for various malignancies. These findings provide a comprehensive overview of the correlation between ferroptosis and cancer, as well as novel valuable insights into the treatment of patients with cancer by targeting ferroptosis.

Importantly, mounting evidence suggests that ferroptosis inhibiters could alleviate the clinical symptoms of intestinal diseases, including intestinal ischemia/reperfusion, IBD, and colorectal cancer [98]. For example, some studies have shown that Liproxstatin-1 can reduce ferroptosis-dependent intestinal I/R injury by promoting GPX4 expression and activating an antagonist of transient receptor potential cation channel subfamily V member 1 (TPRPV1) to inhibit ferroptosis [138]. Studies have shown that treating tubular epithelial cells with erastin induced the expression of ELAL1, a critical regulator in the promotion of ferroptosis and ferritinophagy activation.

## 5. Conclusions and Perspectives

Research on ferroptosis is currently underway. Ferroptosis is a newly identified mode of cell death that is dependent on iron or ROS accumulation. However, many issues regarding ferroptosis remain to be solved. The mitochondria play a central role in oxidative metabolism. Ferroptosis closely interacts with lipid ROS; however, the role of mitochondria in ferroptosis remains unclear. Second, ferroptosis is most commonly observed in cancer cell lines and is observed less frequently in normal cell lines. Moreover, different cancer cells show variable sensitivity to ferroptosis. Therefore, the appropriate inducers, inhibitors, and cell lines should be carefully evaluated in future studies. Third, the features of ferroptosis, such as the process and degree of mitochondrial outer membrane rupture, are still insufficiently characterized. Therefore, the related mechanisms need to be further investigated. Finally, the simple phenomenon of iron-catalyzed increase in ROS levels may not be regarded as ferroptosis. In addition to iron, other metal ions (e.g., copper) can also regulate redox metabolism and similarly affect erastin-induced ferroptosis in HT22 cells [139]. This result suggests that other metals may induce ferroptosis via a regulatory pathway different from iron accumulation. This also implies that iron-induced Fenton reactions are not necessary for ferroptosis. These issues require further in-depth studies.

Many studies have reported that the ferroptosis modulators have great potential for use in the treatment of tumors, and provide a new strategy for eliminating cancer cells. In the future, ferroptosis activators, activator analogs, and inhibitors can be used as drug candidates for cancer treatment. Therefore, the relationship between cancer and ferroptosis needs to be further clarified. Moreover, additional biomarkers, modulators associated with ferroptosis, and the types of tumors related to ferroptosis should be investigated, making this an emerging field of cancer research. As there is still a long way to go in ferroptosis research, an accurate definition of its mechanisms and features is important in ferroptosis-targeting drug development for treating cancer and other diseases.

## Abbreviations

AA, arachidonic acid; AdA, adrenic acid; ACSF2, acyl-CoA synthetase family member 2; ACSL 4, acyl-CoA synthetase long-chain family member 4; AIFM2, apoptosis-inducing factor mitochondria-associated 2; AOA, aminooxyacetic acid; ATP5G3, ATP synthase F0 complex subunit C3; BSO, buthionine sulfoximine; CDKN1A, cyclin-dependent kinase inhibitor 1A; CoQ10, coenzyme Q10; CS, citrate synthase; Cys, cysteine; DFO, deferoxamine; DMT1, divalent metal transporter 1; ETE-PE, eico-sa-tetra-enoyl-phosphatidylethanolamine; Fer-1, ferrostatin-1; Fpn1, ferroportin 1; FSP1, ferroptosis suppressor protein 1; GCL, glutamate-cysteine ligase; GCS, γ-glutamylcysteine synthetase; Gln. glutamine; GLS, glutaminase; Glu, glutamate; Gly, glycine; GPNA, L-g-glutamyl-p-nitroanilide; GPX4, glutathione peroxidase 4; GSH, glutathione; GSSG, glutathione-S-S-glutathione; G6PD, glucose-6-phosphate dehydrogenase; 15-HpETE-PE, 15-hydroperoxy-eicosa-tetra-enoyl-phosphatidylethanolamine; IMCA, 2-imino-6-methoxy-2H-chromene-3-carbothioamide; Lip-1, Liproxstatin-1; LOX, Lipoxygenase; LPCAT3, Lysophosphatidylcholine acyl-transferase 3; LIP, labile iron pool; NAAs, neutral amino acids; NCOA4, nuclear receptor coactivator 4; NDGA, nordihydroguaiaretic acid; Nedd4, Neuronal precursor cell-expressed developmentally downregulated 4; PE, phosphatidylethanolamine; PEBP1, Phosphatidylethanolamine binding protein 1; PepA-Me, Pepstatin A-methyl ester; PGD, phosphoglycerate dehydrogenase; PPAR-γ, Peroxisome proliferator-activated receptor gamma; ROS, reactive oxygen species; RPL8, Ribosomal protein L8; RSL3, RAS-selective lethal small molecule 3; SSZ, sulfasalazine; STEAP3, six transmembrane epithelial antigens of the prostate 3; TAM, tumor-associated macrophages; TfR1, transferrin receptor 1; TTC35, Tetratricopeptide repeat domain 35; TZDs, Thiazolidinediones; VDAC2, Voltage-dependent anion channel 2; VDAC3, Voltage-dependent anion channel 3; α-ketoglutarate (α-KG); β-ME, β-mercaptoethanol.
